# Linking the ovarian cancer transcriptome and immunome

**DOI:** 10.1186/1752-0509-2-2

**Published:** 2008-01-03

**Authors:** Ronald Rapberger, Paul Perco, Cornelia Sax, Thomas Pangerl, Christian Siehs, Dietmar Pils, Andreas Bernthaler, Arno Lukas, Bernd Mayer, Michael Krainer

**Affiliations:** 1 Institute for Theoretical Chemistry, University of Vienna, Währinger Strasse 17, A-1090 Vienna, Austria; 2 University Clinics for Internal Medicine I, Medical University of Vienna, Währinger Gürtel 18-20, A-1090 Vienna, Austria; 3 emergentec biodevelopment GmbH, Rathausstrasse 5/3, A-1010 Vienna, Austria

## Abstract

**Background:**

Autoantigens have been reported in a variety of tumors, providing insight into the interplay between malignancies and the immune response, and also giving rise to novel diagnostic and therapeutic concepts. Why certain tumor-associated proteins induce an immune response remains largely elusive.

**Results:**

This paper analyzes the proposed link between increased abundance of a protein in cancerous tissue and the increased potential of the protein for induction of a humoral immune response, using ovarian cancer as an example. Public domain data sources on differential gene expression and on autoantigens associated with this malignancy were extracted and compared, using bioinformatics analysis, on the levels of individual genes and proteins, transcriptional coregulation, joint functional pathways, and shared protein-protein interaction networks. Finally, a selected list of ovarian cancer-associated, differentially regulated proteins was tested experimentally for reactivity with antibodies prevalent in sera of ovarian cancer patients.

Genes reported as showing differential expression in ovarian cancer exhibited only minor overlap with the public domain list of ovarian cancer autoantigens. However, experimental screening for antibodies directed against antigenic determinants from ovarian cancer-associated proteins yielded clear reactions with sera.

**Conclusion:**

A link between tumor protein abundance and the likelihood of induction of a humoral immune response in ovarian cancer appears evident.

## Background

An intriguing interplay between cancer cells and the body's immune system has been reported, and includes both humoral and cellular pathways [1-3]. Research into links between cancer and the immune system has aimed to acquire further understanding of the mechanisms involved [4], but also addresses applications in diagnostics, disease surveillance, and therapeutic approaches [5-9].

The antibody profile triggered in the course of tumor development (i.e., the spectrum of antibodies directed against tumor-associated components) may be an immunologic fingerprint of the malignant tissue, in turn providing information on disease-associated proteins. Experimental technologies for identification of such autoantigens include display methods such as phage display, serological expression cloning analysis (SEREX), or protein arrays [10-14]. These approaches share the use of selected antigenic determinants to screen for autoantibodies in sera of cancer patients, so that clinically relevant tumor antigens may be indirectly detected. Over the last decade an impressive number of autoantigens have been identified, and SEREX data have been made publicly accessible as a web database [15]. Drawbacks of most display methods, as presently applied, include their limitation to linear epitopes and selection biases arising from various experimental procedures [16]. Protein arrays might overcome both shortcomings, as structural epitopes are amenable to display, and, if processed correctly, may also take post-translational modification into account. Only a limited number of proteins are presently available in arrays, however, and the arrays fail to attain significant and unbiased coverage even of the hitherto-annotated human proteome. Furthermore, aberrant protein modification (such as unusual glycosylation) may be an important source of antigens generating autoantibodies [17], a fact not considered in most screening approaches.

To date, no conclusive explanation has been put forward for why certain proteins become autoantigens in the course of tumor development, whereas others do not. However, autoantibodies are frequently found to react with structures previously not displayed to the mature immune system, such as fetal or viral proteins expressed by malignant cells [18-20]. Further examples include intracellular proteins released by cancer cells into the microenvironment, and the expression of abnormal splice variants [9,16]. Antibodies targeted against mutant proteins are the most direct explanation for the stimulation of an immune response, and the antibodies may well exhibit cross-reactivities with native proteins. Such data have been reported for the proteins encoded by *p53 *(*TP53*) and *CDX2 *[21,22]. It was also shown, however, that autoantibodies against *p53 *protein did not recognize the mutated part of the native protein [16,23,24]. Interestingly, *p53 *mutations frequently cause increased stability of the protein, thereby increasing the relative concentration. This 'concentration effect' leads directly to another proposed cause of autoantigenicity, namely a high (local) abundance of a gene product in cancerous tissue [25]. Thus, significant upregulation of a gene, followed by attainment of a high local concentration of the gene product, may trigger a humoral immune response against such a protein.

In the present study, we tested the hypothesis that the abundance of a protein in cancerous tissue is related to the probability that the protein will induce a humoral immune response. Our analysis is based on data on differential gene expression in ovarian cancer (and the assumed direct relationship between changes in gene expression and changes in effective protein concentration) derived from a meta-analysis including publications comparing normal and cancerous tissue. A second major dataset is composed of public domain ovarian cancer autoantigens as derived by SEREX [15]. These two datasets represented the startpoint for study of the assumed interrelationship between differential gene expression and altered protein abundance on the one hand, and the occurrence of autoantibodies triggered by high abundance of proteins on the other. Because of the excellent availability of both gene expression and SEREX data, ovarian cancer was chosen as a study case. A previous analysis of similarities of gene expression profiles in different tumors as stored in the Cancer Immunome Database [26] showed significant variability between tumors [27], as did comparisons of SEREX datasets for different malignancies. We therefore focused our analysis on one particular tumor, namely ovarian cancer.

We have applied two procedures aimed at unraveling the postulated link between transcriptome and immunome. First, bioinformatics was utilized to compare transcriptional upregulation with experimentally verified autoantigenicity. The work included direct comparison of given gene or protein lists, and exploration of dataset interrelationships at the levels of transcriptional coregulation and protein-protein interaction networks. Second, a selected group of differentially regulated proteins were explicitly tested for autoantigenic propensity in an experimental setting, following in silico antigenicity profiling and candidate epitope selection.

## Results and Discussion

### Analysis workflow

Datasets derived from a literature-based meta-analysis, as well as an experimentally derived list of autoantigens, formed the startpoint of analysis aimed at elucidating any relationship between differential gene expression and protein abundance on the one hand, and the propensity of such proteins to induce humoral immune responses on the other.

In a first analysis, 86 genes showing concerted upregulation in ovarian cancer, as identified by differential gene expression meta-analysis (the Meta-UP dataset), were directly compared to the 81 public domain autoantigens identified by SEREX (SEREX-ovarian dataset), thereby identifying three joint entries. These genes included the *BRCA1 *associated RING domain 1 (*BARD1*), Keratin 8 (*KRT8*), and Mesothelin (*MSLN*). Although this number of conjoint entries is computed as statistically significant (chi^2 ^test) when compared to the number of conjoint members of randomly generated datasets, the number of direct overlaps of upregulated genes and autoantigens is, from a biological viewpoint, less than impressive. We identified only one entry in Meta-DOWN as an autoantigen, namely the platelet-derived growth factor receptor alpha polypeptide (*PDGFRA*). The gene encoding *PDGFRA *has been reported as mutated in cancerous tissue [28,29], providing an explanation for the identified autoantigenic propensity independent of any differential abundance.

Based on this direct comparison of Meta-ALL and SEREX-ovarian datasets, no obvious link between upregulation in gene expression and a subsequent higher abundance of gene products with autoantigenic potential could be derived. However, both datasets most likely provide only a sample of the overall differential gene expression profile or the spectrum of autoantigens.

To overcome the shortcomings of such partial datasets, bioinformatics analysis was performed to bridge the gap between differential gene expression and assumed changes in protein abundance, and presumed autoantigenicity. Concerted expansion of both datasets was undertaken. Procedures used included transcriptional coregulation analysis, studies on conjoint pathways, and exploration of protein interaction networks. The goal of these procedures was to identify conjoint elements amongst transcription factors, pathways, or protein interaction networks, indirectly linking gene expression and autoantigenicity at the level of particular gene lists.

### Differentially expressed genes

The Meta-UP dataset contained 86 genes, ranked by reported literature frequency of differential expression. For example, the gene encoding Mucin was reported as upregulated in seven publications, and as downregulated in one report. As listed in Table 1, some known cancer-associated autoantigens, including several not present in the SEREX-ovarian dataset, were included in the Meta-UP list (these entries are marked in bold), of which the most prominent was Mucin 1 (*MUC1*) [30]. The tumor-associated calcium signal transducer 1 (*TACSTD1*/*Ep-CAM*) [31,32], Mesothelin (*MSLN*) [33], Heat shock protein 90 (*HSPCA*) [34], Keratin 8 (*KRT8*) [35] and *BRCA1*-associated RING domain 1 (*BARD1*) [36] are included.

**Table 1:** List of 86 upregulated genes (Meta-UP) derived from literature meta-analysis. Only genes reported at least twice in the 20 publications reviewed were considered. The total numbers of reported upregulated (U) and downregulated (D) genes are provided. Rows in **bold** indicate protein products that are autoantigens, either reported in the literature or in the SEREX-ovarian dataset.

**Symbol**	**Gene Name (short)**	**U**	**D**	**Symbol**	**Gene Name (short)**	**U**	**D**
***MUC1***	**mucin 1, transmembrane**	7	1	*H2AFZ*	H2A histone family, member Z	2	0
*FOLR1*	folate receptor 1	5	0	*HIST1H2AC*	histone 1, H2ac	2	0
*KLK7*	kallikrein 7	5	0	*HIST1H2BD*	histone 1, H2bd	2	0
***TACSTD1***	**tumor assoc. calcium signal transducer 1**	6	1	*HMGB1*	high mobility group box 1	2	0
*CD47*	CD47 antigen	5	1	*HP*	hyptoglobin	2	0
*CLDN4*	claudin 4	5	1	***HSPCA***	**heat shock 90 kDa protein 1, alpha-like 3**	2	0
*CP*	ceruloplasmin	4	0	*IGLL1*	immunoglobulin lambda-like polypeptide 1	2	0
*CRABP1*	cellular retinoic acid binding protein 1	4	0	*ITGB8*	integrin beta 8	2	0
*KLK6*	kallikrein 6	5	1	*JAG2*	jagged 2	2	0
*PRAME*	preferentially expressed antigen in melanoma	4	0	*KLK5*	kallikrein 5	2	0
*WFDC2*	WAP four disulfide core domain 2	4	0	*KLK8*	kallikrein 8	2	0
*APOE*	apolipoprotein E	3	0	**KRT8**	**keratin 8**	2	0
*CD24*	CD24 antigen	4	1	*LCN2*	lipocalcin 2	3	1
*CD9*	CD9 antigen	3	0	*LOC389831*	hypothetical gene supported by AL713796	2	0
*CKS1B*	CDC28 protein kinase regulatory subunit 1B	3	0	*LU*	lutheran blood group	2	0
*HLA-DPB1*	MHC class II, DP beta 1	3	0	*MAL*	Mal, T-cell differentiation protein	3	1
*KRT18*	keratin 18	3	0	*MAL2*	Mal, T-cell differentiation protein 2	2	0
*PRKCI*	protein kinase C	3	0	*MEIS1*	Meis 1, myeloid ecotropic viral integration site	2	0
*SLPI*	secretory leukocyte protease inhibitor	4	1	*MP14*	matrix metalloprotease 14	2	0
*SPINT2*	serine protease inhibitor	3	0	***MSLN***	**mesothelin**	2	0
*ADAMTS5*	disintegrin-like, metalloprotease	2	0	*MYCl1*	V-myc myelocytomastosis viral oncogene	2	0
*ANK3*	ankyrin 3	2	0	*PAX8*	paired box gene 8	3	1
*ATF3*	activating transcription factor 3	2	0	*PEA15*	phosphoprotein enriched in astrocytes 15	2	0
**BARD1**	**BRCA1 associated RING domain 1**	2	0	*PRKCBP1*	protein kinase C binding protein 1	2	0
*BCL2L1*	Bcl-2 like 1	2	0	*PRKCH*	protein kinase C	2	0
*BMP7*	bone morphogenetic protein 1	3	1	*S100A1*	S100 calcium binding protein A1	3	1
*CD44*	CD44 antigen	2	0	*SCGB2A1*	secretoglobin family 2A member 1	2	0
*CKB*	creatine kinase, brain	2	0	*SCNN1A*	sodium channel, non-voltage gated	2	0
*CLU*	clusterin	2	0	*SDC4*	syndecan 4	2	0
*COL5A1*	collagen type V alpha 1	2	0	*SIAHBP1*	fuse binding protein interacting repressor	2	0
*COL9A2*	collagen type IX alpha 2	2	0	*SLC7A5*	solute carrier family 7, member 5	2	0
*CRABP2*	cellular retinoic acid binding protein 2	2	0	*SOX9*	SRY-box 9	2	0
*D2S448*	melanoma associated gene	2	0	*SPON1*	spondin 1, extracellular matrix protein	2	0
*DPM1*	dolichyl-phosphate mannosyltransferase polypeptide 1	2	0	*VEGF*	vascular endothelial growth factor	2	0
*EDD*	E3 identified by differential display	2	0	*CLDN3*	claudin 3	2	1
*EFNA1*	ephirin A1	3	1	*IGKC*	immunoglobulin kappa constant	2	1
*ELF3*	E74 like factor 3	2	0	*KLK10*	kallikrein 10	2	1
*EYA2*	eyes absent homolog 2 (Drosophila)	2	0	*KRT19*	keratin 19	2	1
*FLJ20171*	hypothetical protein FLJ20171	2	0	*NMU*	neuromedin U	2	1
*FLT1*	fms-related tyrosine kinase 1	2	0	*PTPRF*	protein tyrosine phosphatase, receptor type, F	2	1
*FOXJ1*	forkhead box J1	2	0	*SFN*	stratifin	2	1
*G1P3*	interferon alpha-inducible protein	2	0	*TACSTD2*	tumor associated calcium signal transducer 2	2	1
*GPX3*	glutathione peroxidase 3	2	0	*UBE2C*	ubiquitin conjugating enzyme E2C	2	1

Interestingly, *MUC1 *and *TACSTD1 *were ranked among the top upregulated genes, thus supporting a link between enriched protein concentration and likelihood of autoantigenicity. This finding was further supported by our failure to find known autoantigens in the list of downregulated genes, Meta-DOWN.

### Transcriptional coregulation

Scanning the upstream regions of genes in the Meta-UP dataset resulted in the identification of 32 transcription factors (TFs) with significantly enriched numbers of binding sites when compared to the distribution of transcription factor binding sites found in randomly picked sequences (chi^2 ^test). Among the most significant TFs were those encoded by *E2F*, *HIF1*, *NFY *or *ETS1*, all previously reported to activate a number of genes overexpressed in various cancers [37-40]. In the SEREX-ovarian dataset, six TFs were detected with enriched binding site numbers, namely those encoded by *GATA1*, *MYOD*, *NFKB*, *IK1*, *HIF1 *and *ARNT*. *GATA1 *features in the growth and maturation of a diverse set of tissues. *MYOD *is important in muscle differentiation, and may be involved in inhibition of cell proliferation. *NFKB *is a well-known regulator of cell growth. More interestingly, *HIF1 *is a master regulator coordinating oxygen homeostasis, and allows the cell to survive a lack of oxygen, a relevant situation in malignant tissue.

Genes listed in Meta-UP share significantly more transcription factors than do genes in the SEREX-ovarian dataset. The ovarian cancer gene list derived by our literature meta-analysis thus appears to be under more stringent control than the SEREX-ovarian gene set, exerted by a defined set of transcription factors.

The number of shared motifs in the SEREX-ovarian dataset is lower than that in the Meta-UP collection, although comparable numbers of sequences were analyzed (81 in SEREX-ovarian and 86 in Meta-UP). Joint regulatory control seems more evident in the gene expression dataset and less pronounced in the list given by SEREX-ovarian. Amongst the six TFs enriched in the SEREX-ovarian dataset, however, four, namely *GATA1*, *MYOD*, *IK1 *and *HIF1*, were also found in the Meta-UP dataset, indicating a weak link between the datasets with respect to transcriptional regulation.

### Conjoint pathway analysis

For the approximately 25,000 genes stored in the RefSeq nonredundant sequence database, about 8,000 distinct assignments of gene identifiers and respective pathways are currently defined in the KEGG (Kyoto Encyclopedia of Genes and Genomes) database [41]. For the Meta-UP gene dataset we found 21 distinct pathways, and for the SEREX-ovarian gene dataset 25 such pathways, where each pathway held at least one gene from the given gene lists.

Of this total of 46 pathways, 9 conjoint elements (i.e., pathways containing at least one gene from both datasets) were found. These were cell communication, cytokine-cytokine receptor interaction, TGF-beta signaling, focal adhesion, ECM receptor interaction, adherence junctions, tight junctions, leukocyte transendothelial migration, and regulation of the actin cytoskeleton. To determine the statistical significance of this finding, we utilized a dataset generation procedure to derive 1,000 random gene lists holding the same number of entries (i.e., 86 for the Meta-UP controls and 81 for the SEREX-ovarian controls). For each of the randomly composed datasets, the numbers of conjoint pathways were computed. This procedure resulted in a normal distribution of conjoint pathways, showing a mean of 6.7 pathways jointly held by two randomly generated datasets, compared to the nine conjoint pathways populated by Meta-UP and SEREX-ovarian genes. Therefore, no significant enrichment of conjoint pathways between Meta-UP and SEREX-ovarian datasets was noted.

### Protein-protein interactions and networks

**Figure 1:**
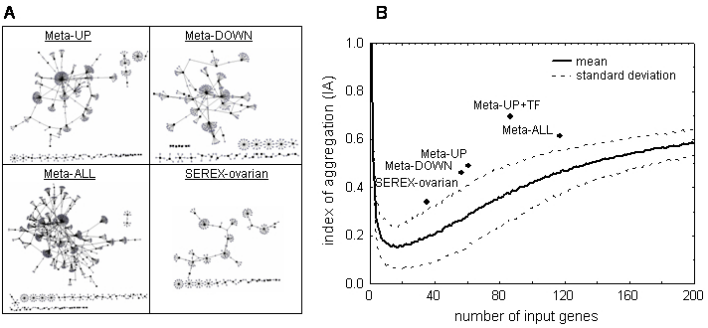
Protein networks based on protein-protein interaction data in OPHID. **A**: Individual interaction networks of Meta-UP, Meta-DOWN, Meta-ALL and SEREX-ovarian datasets as visualized using ProteoLens . **B**: The indices of aggregation (IA) for the given datasets with respect to the IA of ensembles of randomly generated datasets used as references are shown (means and standard deviations).

We expanded the lists of genes encoded by the datasets using nearest neighbor expansion based on OPHID protein-protein interaction data [42]. This approach is based on the rationale that proteins showing differential abundance might show interactions with other proteins embedded in the same functional context (i.e., their nearest neighbors). The resulting interaction networks, as well as their aggregation indices [27] with respect to a reference curve based on random gene selections, are shown in Figure 1.

For Meta-UP, the procedure resulted in networks consisting of 476 nodes and 477 edges. The largest subnetwork was composed of 329 nodes and 354 edges. Thirty of the 61 detected genes stored in the Meta-UP dataset were found in the largest subnetwork, resulting in an Index of Aggregation (IA) of 0.49. Corresponding IAs for the Meta-DOWN, Meta-ALL, and SEREX-ovarian datasets were 0.46, 0.61, and 0.34, respectively.

To permit statistical evaluation of these aggregation indices, a reference curve holding mean IAs and standard deviations for 1,000 randomly generated datasets containing 5–200 genes was computed. This procedure is based on the following rationale: Datasets derived by a systematic selection procedure (such as differential gene expression analysis) may be characterized by aggregation indices clearly exceeding the IAs of randomly generated datasets if they show IAs at least one standard deviation above the mean IA of reference datasets with equal numbers of elements. We additionally constructed an interaction network based on genes stored in Meta-UP including (previously predicted) enriched transcription factors. The resulting network showed an IA of 0.697, and this was highly significant when compared to the distribution obtained from random datasets. Despite putative biases in this dataset generation procedure (well-studied proteins reported, for example, in the context of cancer, show better coverage in protein interaction data), all datasets exhibited internal structures on the level of protein-protein interaction networks. However, datasets derived by differential gene expression clearly showed higher protein-protein interaction network complexity than did the SEREX-ovarian dataset.

Direct comparison of gene identifiers in the Meta-UP and SEREX-ovarian datasets resulted in three joint entries, as noted above. The number of genes shared between the datasets after performing nearest neighbor expansion at the level of protein-protein interactions is presented in Table 2.

**Table 2:** Number of conjoint genes found by directly comparing gene identifiers as stored in primary datasets, and by comparing datasets expanded by transcription factors and nearest neighbor protein-protein interactions. Meta-UP, Meta-ALL, and SEREX-ovarian are the original source datasets. Meta-UP+TFs represents the upregulated genes of Meta-ALL additionally enriched by identified transcription factors. Meta-UP, expanded, is the original Meta-UP gene list expanded by nearest neighbor protein-protein interactions. Meta-UP+TFs, expanded, additionally includes associated transcription factors. Meta-ALL, expanded, and SEREX, expanded, are the original datasets expanded by nearest neighbor protein-protein interactions.

	**Meta-UP (86)**	**Meta-ALL (192)**	**SEREX (81)**
*Meta-UP*	-	-	3
*Meta-ALL*	-	-	4
*SEREX-ovarian*	3	4	-
*Meta-UP+TFs*	-	-	3
*Meta-UP, expanded*	-	88	5
*Meta-UP+TFs, expanded*	-	92	6
*Meta-ALL, expanded*	-	-	6
*SEREX-ovarian, expanded*	6	9	-

As clearly indicated by respective pairwise comparisons of original datasets and expanded datasets, the overlap between gene expression and SEREX-ovarian datasets is still minor. The Meta-UP dataset including associated transcription factors and nearest neighbors at the level of protein-protein interaction resulted in a list of 756 proteins of which, in addition to the three entries already identified via direct comparison, only three additional entries (*STUB1*, *UBE3A*, *ACVR2B*) were also listed in the SEREX-ovarian dataset.

Information derived by comparison of gene expression and SEREX-ovarian datasets, although expanded by coregulation and network analysis, indicated no major link between local abundance of a protein and its potential for autoantigenicity, at least at the level of given datasets. Differential gene expression appears to affect functional dependencies identified on the basis of transcription factors involved in regulation, and protein interaction partners, whereas particular autoantigens appear to be random selections from the human proteome.

To ascertain if the given datasets were biased selections, therefore failing to provide significant overlaps, or if abundance (as estimated based on differential gene expression) might not be linked to increased propensity for autoantigenicity at all, explicit experimental testing was performed.

### Immunogenicity profiling

To finally test whether upregulation might increase the autoantigenic potential of a protein (i.e., that a break in immune tolerance might result from a local concentration effect), in silico immunogenicity screening of proteins encoded by upregulated genes was performed. Identified candidate epitopes were subsequently tested experimentally for identification of reactive antibodies in ovarian cancer patient sera.

We included all sequences from the Meta-UP dataset which were reported as upregulated in at least three publications (as distinct from the two reports required for membership in the Meta-UP dataset used in earlier analyses). We further included three sequences listed in Meta-UP which were also reported in the SEREX-ovarian dataset. Further, the top five upregulated genes of the two gene expression raw datasets at hand were selected [43,44], as was *TP53*, a well-known cancer autoantigen [24,34], serving as a positive control.

In total, 61 proteins were identified for virtual immunogenicity profiling by our selection procedure. We utilized E-Score to identify candidate linear epitopes on the proteins. E-Score uses the primary sequence of a protein and combines structural features via 2D/3D structure prediction and solvent accessibility analysis with a neural network-based immunogenicity scoring function. The outputs of the scoring procedure are linear candidate B-cell epitopes (with a mean length of 17 aa). Thirty-one of the 61 proteins gave promising immunogenicity profiles; the remaining 30 proteins were not analyzed further. From the 31 proteins providing good immunogenicity profiles, 88 individual candidate epitopes were selected, synthesized, and experimentally used. As a reference dataset, 88 candidate epitopes from 31 proteins randomly picked from the Meta-DOWN dataset were selected.

### Experimental epitope verification

The peptides were screened in a peptide-ELISA setting, mounting biotinylated candidate epitopes on streptavidin-coated microtiter plates. Peptides were then screened utilizing sera from ovarian cancer patients and sera from healthy controls to determine the prevalence of antibodies which showed reactivities with the peptides. All 88 peptides of each dataset were screened using a tumor sera pool (composed of 20 sera) obtained from ovarian cancer patients and a reference sera pool (composed of 10 sera) from non-cancer female patients. Table 3 lists the clinical characteristics of patients whose sera were used.

**Table 3:** Characteristics and clinical parameters of the 20 ovarian cancer patients whose sera were used in this study. All patients had adenocarcinomas.

**Sample No.**	**Year of birth**	**Year of diagnosis**	**Year of sample collection**	**Sample taken after relapse**	**Grade**	**FIGO Stage**
1	1934	1998	2004	Yes	3	Ic
2	1928	2002	2004	Yes	3	IIIc
3	1941	2002	2005	Yes	3	IIIA
4	1937	2004	2004	No	-	-
5	1931	1997	-	-	2	IIIc
6	1954	2002	2005	Yes	3	IV
7	1955	2002	2005	Yes	3	IIIc
8	1937	1996	2005	Yes	X	X
9	1937	1994	2005	Yes	3	IIIc
10	1956	2001	2005	Yes	3	IIIc
11	1941	2002	-	-	3	IIIc
12	1942	1996	2004	-	3	Iic
13	1940	2004	2004	No	2	IIIa
14	1923	1997	2004	-	2	Ia
15	1933	2004	2004	No	1	IV
16	1941	2004	2005	-	2	IIIc
17	1947	2002	2004	No	-	IIIc
18	1941	1995	2005	Yes	3	Ic
19	1928	1999	2004	Yes	3	III
20	1935	2002	2005	Yes	3	IIIc

**Figure 2:**
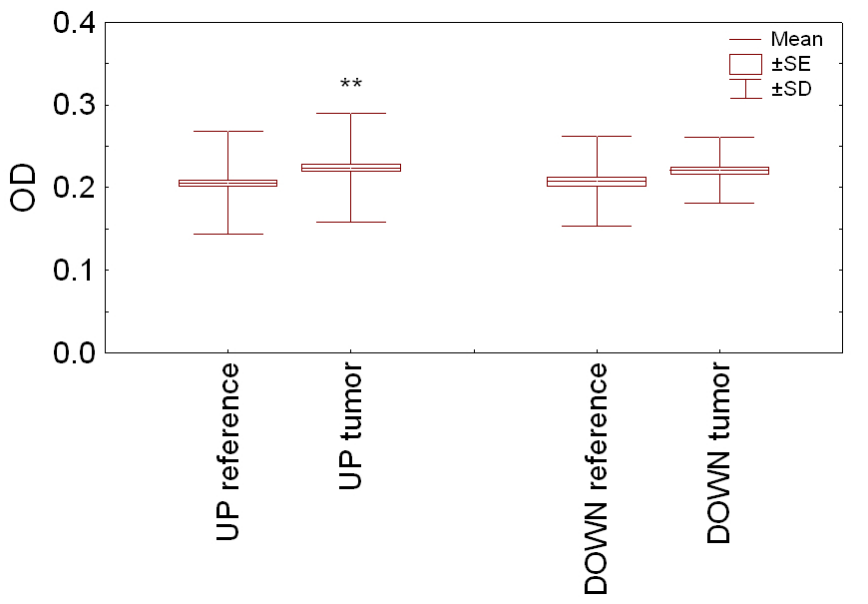
Box-plots giving means, errors of means, and standard deviations of ELISA signal intensities from the tumor sera pool (tumor) and the reference sera pool (reference), using equal numbers of antigenic peptides from Meta-UP (UP) and Meta-DOWN (DOWN) proteins. The OD values are ELISA signal readouts. A double asterisk indicates a highly significant difference based on Student's *t*-test (*p *= 0.0011).

Figure 2 gives the raw ELISA signal intensity distributions for all peptides tested using the tumor sera pool (20 samples) and the reference sera pool (10 samples), and compares candidate epitopes selected from upregulated (UP) and downregulated (DOWN) genes.

**Figure 3:**
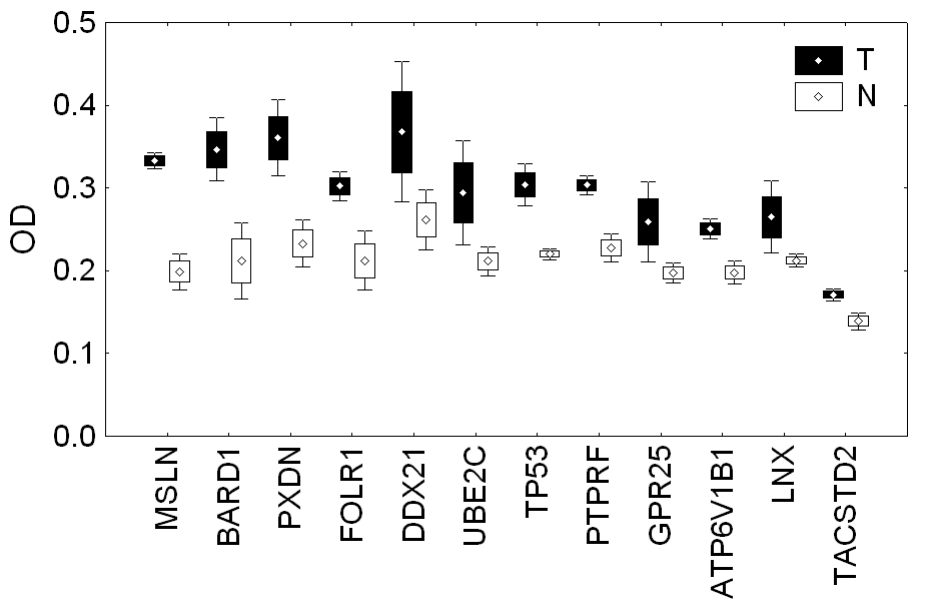
Box-plots giving means, errors of means, and standard deviations of triplicate measurements of ELISA signals (OD, optical density) for the 12 proteins exhibiting the highest signal differences when a tumor sera pool (20 sera) and a reference sera pool (10 sera) were compared (**A**), and the corresponding data for the remaining 19 proteins (**B**). Where more than one epitope was tested for a given protein the signal based on the epitope showing strongest reactivity is provided. Black box-plots indicate tumor sera reactivity and white box-plots give reference sera reactivity. Each protein is named from its gene symbol.

ELISA signal intensities were compared between the sample groups using Student's *t*-test, and a significant difference was found when reactivities of healthy and diseased sera on epitopes derived from upregulated genes were compared (*p *= 0.0011). In contrast, no significant difference was observed between tumor and reference sera pools reacting with epitopes derived from Meta-DOWN proteins. When all candidate epitopes were included in the statistical analysis, however, the absolute difference between reference and tumor sera was small also for Meta-UP candidate epitopes. First, it is likely that not all upregulated genes provide proteins triggering autoantibody production because of various host factors. Second, the in silico epitope prediction may have missed important immunogenic determinants. Figure 3A shows sera reactivity of tumor and reference sera for the 12 of the 31 proteins showing the largest reactivity differences; Figure 3B provides the data for the remaining 19 proteins.

Among the upregulated and most-reactive proteins, four well known autoantigens reported in various tumors were found, namely MSLN (mesothelin), BARD1 (BRCA1 associated RING domain 1), LNX (ligand of numb-protein X 1) and TP53 (tumor protein 53), which we included as an internal control. Interestingly, a series of receptor molecules was identified as potential autoantigenic components in ovarian cancer. These included the folate receptor (FOLR1), the protein tyrosine phosphatase receptor type F (PTPRF), the G protein-coupled receptor 25 (GPR25), ATPase subunit B1 (ATP6V1B1), and the tumor-associated calcium signal transducer 2 (TACSTD2). In particular, the identification of TACSTD2 is interesting. This protein belongs to a family including at least two Type I membrane proteins, one of which is the widely investigated TACSTD1, better known as epithelial cell adhesion molecule (Ep-CAM). Autoantibodies in the sera of tumor patients, in particular those with ovarian cancer, have been reported for Ep-CAM [31].

**Figure 4:**
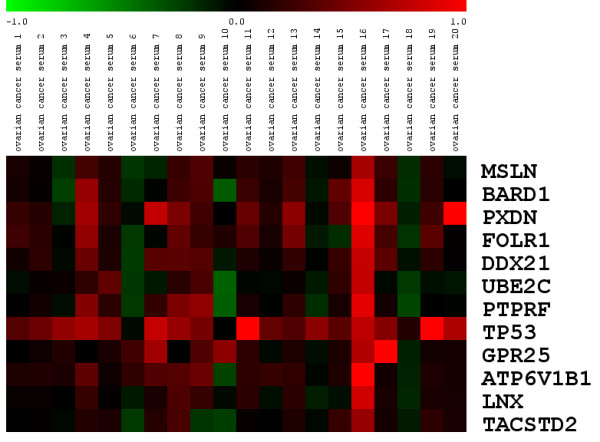
Heat-map representation of ELISA signal intensities for the 12 most reactive epitopes of 12 individual proteins screened with 20 individual ovarian cancer patient sera. Signals are color coded for the interval [-1,1] and represent the log_2_-transformed differences between the ELISA signals using tumor serum and signals derived using a control peptide as a background reference. Red coloring indicates increased reactivity of an individual tumor serum.

Figure 4 shows the reactivities of epitopes selected from the 12 proteins shown in Figure 3A, at the level of individual tumor patient sera. ELISA data are given as log_2_-transformed differences between individual tumor sera signals and signals derived using a control peptide as a background reference. An attempt to link available clinical data (Table 3) with sera reactivities (Figure 4) did not show any significant association.

It is important to note that the screening results given in Figures 3 and 4 are based on data indicating upregulation at the level of differential gene expression, not necessarily correlating with changes in protein abundance. We therefore scanned both the literature and the human protein atlas [45] for indications of increased protein abundance of the sequences included in Figure 3A and Figure 4. For 5 (MSLN, BARD1, FOLR1, UBE2C, TP53) of the 12 proteins, clear evidence for increased protein concentrations in ovarian cancer tissue compared to normal ovarian epithelium is available [46-50], supporting the link between upregulation at the gene expression level and increased protein abundance.

## Conclusion

In our work, ovarian cancer was used as a test system to investigate whether high expression of cancer-associated proteins is linked to an increased propensity towards autoantigenicity in the context of a humoral immune response. The startpoint of our analysis was the integration of datasets derived from public domain differential gene expression analyses, as well as reported SEREX data on ovarian cancer autoantigens.

The meta-analysis of 20 publications showed differential gene expression of more than 1,700 genes. Only 192 genes, however, were reported in more than one publication, clearly reflecting shortcomings in experimental procedures and downstream processing of differential gene expression analyses, as well as the heterogeneous nature of this tumor entity [51]. Amongst the 192 genes, 86 were reported as upregulated (Meta-UP) and 106 as downregulated (Meta-DOWN).

In a first step, we explicitly compared our literature-derived Meta-UP gene set with the SEREX-defined autoantigens for ovarian cancer (SEREX-ovarian; 81 genes). Three conjoint genes were identified, whereas only one protein represented in Meta-DOWN (106) was also present in the SEREX-ovarian dataset. Additionally, a set of literature-reported cancer autoantigens was found in Meta-UP but not in Meta-DOWN, including Mucin 1 (*MUC1*), the tumor associated calcium signal transducer 1 (*TACSTD1*), and the heat-shock protein 90 (*HSPCA*) [7,34,52]. These examples indicated a link between gene overexpression and protein autoantigenic potential, whereas a direct comparison between gene expression data and SEREX-ovarian genes did not suggest such a correlation.

Presentation to the humoral immune system is mandatory for triggering the production of antibodies, a process facilitated either by antigen presenting cells, or occurring via direct antigen access. Consequently, autoantigens may accumulate in the extracellular space or cell wall, or may be secreted. However, SEREX-defined gene products show a tendency towards nuclear location. One explanation for this finding might be cell breakage and consequent necrosis in the course of tumor progression.

In any case, both datasets most likely represented but small selections of differentially expressed genes or autoantigens. In light of this, transcriptional coregulation analysis, pathway analysis, and protein-protein interaction analysis were performed to identify indirect links between the given datasets. On the level of transcriptional coregulation, we identified a series of well-known, cancer-associated TFs as over-represented in Meta-UP, significantly overlapping with enriched TFs also found in the common cancer profile dataset of Rhodes *et al.* ([53], data not shown). A smaller number of TFs characteristic of the SEREX-ovarian dataset was identified, but, amongst the six TFs found, four were also characteristic of the Meta-UP gene set.

After protein-protein interaction analysis, interaction networks derived from both the SEREX-ovarian and Meta-UP datasets showed increased IAs; however, even after a first neighbor expansion, the overlap between the datasets did not increase significantly. The protein-protein interaction analysis revealed a systematic logic in and inherent complexities of both the Meta-UP and SEREX-ovarian datasets. However, the datasets could not be convincingly linked via one-neighbor extension. Weak correlation was also found when searching for conjoint KEGG pathways [41]. Nine of 46 pathways were identified as jointly populated by entries from the Meta-UP and SEREX-ovarian datasets.

Based on these results, a tight linkage between high abundance as identified by differential gene expression analysis, and autoantigenic potential as found by membership of the SEREX-ovarian dataset, could not be shown. The gene expression dataset on its own appears conclusive, exhibiting a significant number of joint transcription factors, good integration with KEGG pathways, and a high IA at the level of protein-protein interactions. The SEREX-ovarian dataset showed a less integrated picture, but is clearly set apart from randomly selected gene lists. The true set of autoantigens might therefore still be linked to concerted intracellular events, not necessarily coupled to massive changes in expression, in contrast to a profile appearing as random, as would result if cell breakage and necrosis were the sole sources triggering a humoral response against intracellular proteins.

**Figure 5:**
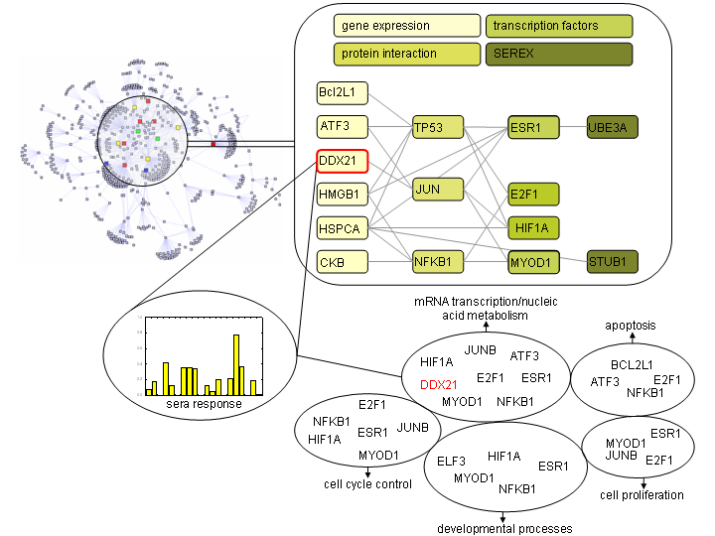
Data integration scheme: Protein-protein interaction networks unravel the link between gene expression and SEREX-ovarian data, via identified transcription factors. One of the newly identified autoantigens, *DDX21*, is included. The protein network was generated using genes identified by Meta-UP and SEREX-ovarian dataset searches. Genes shown in the upper right present a sample of the network, linking the various data sources to the newly identified autoantigen DDX21. In the lower left of the Figure, ELISA signal intensities of the 20 individual ovarian cancer sera tested on DDX21 are given as bar plots. Genes involved can be further grouped using gene ontology terms, showing typical gene categories involved in cancer.

To further study potential links between protein overexpression and autoantigenicity, we explicitly tested 31 proteins showing strong upregulation in an experimental setting. After identifying candidate epitopes on these proteins with an in silico prediction procedure, we conducted ELISA screenings using sera from ovarian cancer patients and from healthy subjects. Although reactivities varied notably amongst different patient sera, we successfully identified 18 epitopes on 12 proteins. Proteins were ranked with respect to sera reactivities. The well-described autoantigen *TP53 *was found amongst the top-ranked proteins, supporting the validity of our approach. Even higher serum reactivity than found for *TP53 *was observed for six proteins, namely MSLN, BARD1, PXDN, FOLR1, DDX21 and UBE2C. Proteins MSLN and BARD1 are well-known autoantigens of ovarian cancer and have also been found by SEREX. Protein PXDN is a melanoma-associated protein and the ubiquitin-conjugating enzyme E2C (UBE2C) is believed to play a role in tumor progression [54,55]. Folate receptor 1 has often been reported as significantly upregulated in ovarian cancer and is also known as ovarian carcinoma-associated antigen [49]. To date, no link to cancer or to a humoral autoimmune response has been reported for the RNA helicase DDX21, indicating that this is a newly-described autoantigen. In contrast to results obtained by comparing the Meta-UP and SEREX-ovarian datasets, our experimental data point towards a link between protein overexpression and autoantigenicity. Following an integrated analysis approach, diverse links between the various layers of differential gene expression, transcriptional coregulation, protein-protein interactions, and autoantigenicity, can be drawn, as schematically represented in Figure 5.

Figure 5 provides a detailed view of selected protein-protein interactions as embedded in a protein interaction graph when all elements of Meta-UP, SEREX-ovarian, and identified transcription factors are included. Starting with differentially expressed genes (including the newly identified autoantigen *DDX21*) and central control elements such as *TP53, JUNB *and *NFKB*, further transcription factors are linked, finally also including selected elements from the SEREX-ovarian dataset. Analysis of the gene ontologies of involved elements results in conclusive functional groups, namely apoptosis, cell proliferation, cell cycle control, nucleic acid metabolism, and developmental processes. These clearly link the integrated network of differentially expressed genes, transcription factors, and autoantigens, to molecular processes associated with cancer development and progression. Our data indicate that changes in gene expression are closely associated with processes occurring in malignant cells, and some proteins relevant in this context appear to exhibit increased autoantigenic propensities. A wide range of additional effects come into play, however, including only a partial correlation between upregulation of gene expression and protein abundance, variations in the efficiencies of epitopes in triggering generation of antibodies, and the general immune status of tumor patients. These conditions may be responsible for the heterogeneous autoantibody spectrum found in cancer patients. The use of available data on differential gene expression as a startpoint for the screening of large numbers of patient sera may, however, be a valuable strategy for identifying autoantigens prevalent in cancer, concomitantly contributing to the establishment of novel immunotherapies.

## Methods

### Datasets

Peer-reviewed publications on ovarian cancer gene expression analysis were identified in PubMed [56], accessed in June 2005, applying a keyword search (ovarian cancer, gene expression, microarrays). All array platform technologies were included. In total, 25 publications were retrieved, and 20 papers published from 1999–2005 were gene expression studies comparing ovarian cancer tumor tissue with either healthy tissue or ovarian epithelial cell lines as references. This list of publications was included in our meta-analysis and is presented in Table 4. From these papers, differentially expressed genes (given by their GeneIDs) were manually extracted and used for subsequent analysis.

**Table 4:** Twenty publications (1999–2005) comparing ovarian cancer tissue with healthy ovarian tissue or ovarian cell lines, utilizing differential gene expression. Genes reported as differentially expressed represent the Meta-ALL dataset. Papers are listed by first author only.

**Publication – journal**	**Reference**
Zhang, Int. J. Gynecol. Cancer (2005)	[69]
DeCecco, Oncogene (2004)	[70]
Donninger, Oncogene (2004)	[71]
Lancaster, Soc. Gynecol. Investig. (2004)	[72]
Lee, Int. J. Oncol. (2004)	[73]
Santin, Int. J. Cancer (2004)	[74]
Collins, Int. J. Mol. Med. (2004)	[75]
Hibbs, Am. J. Pathol. (2004)	[76]
Adib, Br. J. Cancer (2004)	[77]
Zorn, Clin. Cancer. Res. (2003)	[78]
Matei, Oncogene (2002)	[79]
Sawiris, Cancer Res. (2002)	[80]
Welsh, Proc. Natl. Acad. Sci. USA (2001)	[44]
Shridhar, Cancer Res. (2001)	[81]
Hough, Cancer Res. (2001)	[82]
Mok, J. Natl. Cancer Inst. (2001)	[83]
Hough, Cancer Res. (2000)	[84]
Ismail, Cancer Res. (2000)	[85]
Ono, Cancer Res. (2000)	[86]
Schummer, Gene (1999)	[87]

Based on the gene identifiers from the 20 publications, a non-redundant gene set was generated. In total, more than 1,700 unique genes were reported as being differentially expressed when healthy and diseased cells were compared. To account for biological and technical variances inherent in this meta-analysis, only genes reported in more than one publication as differentially regulated were kept in our core dataset. This led to a list of 192 genes (dataset Meta-ALL). Amongst these 192 unique genes, 86 were reported as significantly upregulated (dataset Meta-UP) and 106 genes were reported as significantly downregulated (Meta-DOWN). The list of upregulated genes as derived by our analysis is given in Table 1. Besides the Meta-ALL gene list, a publicly available dataset holding cancer autoantigens as identified by SEREX was retrieved from the Cancer Immunome Database [26]. A database search for the tissue 'ovary' (in December 2005) revealed 81 non-redundant genes represented by their GeneIDs. This dataset was termed SEREX-ovarian.

### Consensus analysis of given gene tables

A range of bioinformatics analyses were conducted, utilizing the datasets derived from the meta-analysis covering differential gene expression and SEREX. Procedures included identification of joint elements via direct comparison of GeneIDs, but focused, in particular, on the level of indirect control by analyzing transcriptional coregulation, concerted pathways, and protein-protein interaction networks. Given genes (and the proteins they encode) may show no direct overlap, but may still be under joint control on the transcriptional level, or might populate the same protein pathways.

For identification of transcriptional coregulation, a transcription factor binding site (TFBS) analysis was performed. First, the regulatory regions of genes stored in the datasets Meta-UP and SEREX-ovarian were extracted. Regulatory regions were identified utilizing the CONFAC tool for deriving human-to-mouse orthologous promoter sequences within 2,000 bp upstream of transcription start sites [57]. Single TFBSs were predicted in these regulatory regions using known binding site motifs as stored in the TRANSFAC database (matrix and core similarity values of 0.85 and 0.95 were employed) [58]. A Mann-Whitney U test was employed to identify TFs with enriched numbers of binding sites in the regulatory regions of a given gene set (i.e., the Meta-UP and SEREX-ovarian datasets) when compared to randomly selected reference datasets. For identification of joint promoter modules (as combinations of TFBSs) a genetic algorithm was applied as described in Perco *et al.*[59]. The outputs of coregulation analysis are lists of transcription factors and combinations of transcription factors enriched in Meta-UP or SEREX-ovarian, or jointly enriched in both datasets.

Pathway analysis of given datasets was performed utilizing the KEGG database [41] to unravel functional protein networks covered by the given gene lists. All known regulatory networks were retrieved from the public domain data repository, provided that such individual networks held at least one element of a given dataset. Datasets were then compared at the level of pathways populated by the different gene datasets. The significance of findings was evaluated by comparison of the number of conjoint pathways found for the given datasets with the number of conjoint pathways found using randomly generated datasets of the same sizes.

Human protein-protein interactions (PPIs) as provided by OPHID were used to determine the interactions of proteins encoded by genes stored in the different datasets [42]. The goal of this procedure was the construction of protein-protein interaction graphs and a subsequent comparison of subgraphs identified through the use of particular gene expression and autoantigen datasets. The high quality interaction subsets provided by BIND [60], MINT [61], MIPS [62], HPRD [63], RikenDIP and RikenBIND [64], in total representing 20,289 pairwise protein interactions, were used. PPI networks were generated using the nearest neighbor expansion method as proposed by Chen and colleagues [65]. Levels of aggregation and complexities of derived interaction networks were quantified by computing IA values [27], which are the percentages of selected nodes in the largest subnetwork with respect to all selected nodes in a network generated from a given gene list. The IAs of networks derived from given gene sets were compared with the IAs of randomly selected gene lists to identify statistically significant levels of protein interaction in gene expression and autoantigen datasets.

The integrated application of differential gene expression, coregulation analysis, and protein network exploration, based on the datasets retrieved, was the basis for comparison of the ovarian cancer transcriptome and immunome. Further details on our methodological workflow are outlined in Perco *et al.*[66].

### In silico and experimental antigenicity analysis

Consensus analysis of given gene lists focused on identification of joint elements, as well as on elucidation of indirect control, by considering individual elements in the lists. For identification of *novel *autoantigens in a given gene expression list, information on subcellular location and on potential antigenic determinants (epitopes) of candidate proteins was derived. Subcellular locations of proteins were predicted using PSORT for eukaryotic sequences [67,68]. Linear B-cell epitopes were predicted using the antigenicity classification function E-score (emergentec biodevelopment, Vienna, Austria). E-scores are based on sequence descriptors derived from extended, experimentally verified B-cell epitope and reference datasets, combined in a neural network-type classification function.

Predicted immunogenic determinants on disease-associated proteins were subjected to experimental verification. Sera from 20 ovarian cancer patients and from 10 presumably healthy subjects were used for identification of reactive antibodies prevalent in given sera. Usage of sera was approved by the Ethical Committee of the Medical University of Vienna. Confidentiality of study subjects was assured by sample coding.

Candidate epitopes (17 aa in length) were synthesized (Mimotopes Pty Ltd., Clayton Victoria Australia) with N-terminal biotin, followed by a four aa spacer sequence (SGSG). For experimental screening, streptavidin-coated 96-well microtiter plates (Mimotopes) were blocked with 200 μl/well of 2% (w/v) bovine serum albumin (Sigma-Aldrich, St. Louis, MO) in PBST (PBS [0.1 M sodium phosphate, 0.15 M NaCl, pH 7.0] + 0.1% [v/v] Tween 20) overnight at 4°C. Subsequently, the wells were washed four times with PBST and incubated with the biotinylated peptides for 2 h at room temperature. Blank wells were incubated with PBST in the absence of peptides. The wells were washed four times with PBST, and 100 μl of sera diluted 1:400 or 1:800 was added to each well. Incubation for 2 h at room temperature followed. After further washing, antibody binding was detected using 100 μl/well of alkaline-phosphatase conjugated goat anti-human IgG (BETHYL Laboratories, Inc., Montgomery, U.S.) diluted in blocking buffer (1:1000), with incubation for 1 h. After 6 washes with PBST, 200 μl of a 1.0 mg/ml p-nitrophenylphosphate substrate solution in 0.2 M Tris-buffer (Sigma-Aldrich) was added to each well. Absorbance was measured on a BDSL Immunoskan PLUS spectrophotometer at 405 nm after 90 min. All measurements were performed in triplicate. Control wells for testing the secondary antibody, as well as a reference peptide serving as a negative control, were included [18].

### Patient sera

Ovarian cancer and reference sera were collected at the Medical University of Vienna after receiving patient consent. Twenty individual sera of patients with diagnosed ovarian cancer, at different cancer stages (Table 3), and 10 reference sera from healthy women aged 20–30 years, with no indications of cancer or ovarian pathology, were collected.

## Authors' contributions

RR carried out the bioinformatics analysis, performed the protein antigenicity profiling, and drafted the manuscript. PP performed the identification of transcription factor binding sites. CS conducted the pathway analysis and AB performed the protein-protein interaction analysis. TP and CS were responsible for the experimental ELISA screenings. DP collected data on gene expression and sera reactivities.

AL participated in the design of the study and performed the statistical analysis. MK and BM conceived the study, participated in the design and coordination, and prepared the final manuscript.

## References

[B1] Ichiki Y, Takenoyama M, Mizukami M, So T, Sugaya M, Yasuda M, So T, Hanagiri T, Sugio K, Yasumoto K (2004). Simultaneous cellular and humoral immune response against mutated p53 in a patient with lung cancer. J Immunol.

[B2] Jager E, Chen YT, Drijfhout JW, Karbach J, Ringhoffer M, Jager D, Arand M, Wada H, Noguchi Y, Stockert E, Old LJ, Knuth A (1998). Simultaneous humoral and cellular immune response against cancer-testis antigen NY-ESO-1: definition of human histocompatibility leukocyte antigen (HLA)-A2-binding peptide epitopes. J Exp Med.

[B3] Sreekumar A, Laxman B, Rhodes DR, Bhagavathula S, Harwood J, Giacherio D, Ghosh D, Sanda MG, Rubin MA, Chinnaiyan AM (2004). Humoral immune response to alpha-methylacyl-CoA racemase and prostate cancer. J Natl Cancer Inst.

[B4] de Visser KE, Eichten A, Coussens LM (2006). Paradoxical roles of the immune system during cancer development. Nat Rev Cancer.

[B5] De Groot AS (2006). Immunomics: discovering new targets for vaccines and therapeutics. Drug Discov Today.

[B6] Finn OJ (2005). Immune response as a biomarker for cancer detection and a lot more. N Engl J Med.

[B7] Hirasawa Y, Kohno N, Yokoyama A, Kondo K, Hiwada K, Miyake M (2000). Natural autoantibody to MUC1 is a prognostic indicator for non-small cell lung cancer. Am J Respir Crit Care Med.

[B8] Takeda A, Shimada H, Nakajima K, Suzuki T, Hori S, Hayashi H, Arima M, Kouzu T, Ochiai T, Isono K (2000). Impact of circulating p53 autoantibody monitoring after endoscopic resection in mucosal gastric cancer. Endoscopy.

[B9] Wang X, Yu J, Sreekumar A, Varambally S, Shen R, Giacherio D, Mehra R, Montie JE, Pienta KJ, Sanda MG, Kantoff PW, Rubin MA, Wei JT, Ghosh D, Chinnaiyan AM (2005). Autoantibody signatures in prostate cancer. N Engl J Med.

[B10] Chen YT, Gure AO, Scanlan MJ (2005). Serological analysis of expression cDNA libraries (SEREX): an immunoscreening technique for identifying immunogenic tumor antigens. Methods Mol Med.

[B11] Minenkova O, Pucci A, Pavoni E, De Tomassi A, Fortugno P, Gargano N, Cianfriglia M, Barca S, De Placido S, Martignetti A, Felici F, Cortese R, Monaci P (2003). Identification of tumor-associated antigens by screening phage-displayed human cDNA libraries with sera from tumor patients. Int J Cancer.

[B12] Mischo A, Wadle A, Watzig K, Jager D, Stockert E, Santiago D, Ritter G, Regitz E, Jager E, Knuth A, Old L, Pfreundschuh M, Renner C (2003). Recombinant antigen expression on yeast surface (RAYS) for the detection of serological immune responses in cancer patients. Cancer Immun.

[B13] Sioud M, Hansen MH (2001). Profiling the immune response in patients with breast cancer by phage-displayed cDNA libraries. Eur J Immunol.

[B14] Zangar RC, Varnum SM, Bollinger N (2005). Studying cellular processes and detecting disease with protein microarrays. Drug Metab Rev.

[B15] Sahin U, Tureci O, Pfreundschuh M (1997). Serological identification of human tumor antigens. Curr Opin Immunol.

[B16] Anderson KS, LaBaer J (2005). The sentinel within: exploiting the immune system for cancer biomarkers. J Proteome Res.

[B17] Mintz PJ, Kim J, Do KA, Wang X, Zinner RG, Cristofanilli M, Arap MA, Hong WK, Troncoso P, Logothetis CJ, Pasqualini R, Arap W (2003). Fingerprinting the circulating repertoire of antibodies from cancer patients. Nat Biotechnol.

[B18] Humer J, Waltenberger A, Grassauer A, Kurz M, Valencak J, Rapberger R, Hahn S, Lower R, Wolff K, Bergmann M, Muster T, Mayer B, Pehamberger H (2006). Identification of a melanoma marker derived from melanoma-associated endogenous retroviruses. Cancer Res.

[B19] Lu M, Nakamura RM, Dent ED, Zhang JY, Nielsen FC, Christiansen J, Chan EK, Tan EM (2001). Aberrant expression of fetal RNA-binding protein p62 in liver cancer and liver cirrhosis. Am J Pathol.

[B20] Sauter M, Roemer K, Best B, Afting M, Schommer S, Seitz G, Hartmann M, Mueller-Lantzsch N (1996). Specificity of antibodies directed against Env protein of human endogenous retroviruses in patients with germ cell tumors. Cancer Res.

[B21] da Costa LT, He TC, Yu J, Sparks AB, Morin PJ, Polyak K, Laken S, Vogelstein B, Kinzler KW (1999). CDX2 is mutated in a colorectal cancer with normal APC/beta-catenin signaling. Oncogene.

[B22] Wicking C, Simms LA, Evans T, Walsh M, Chawengsaksophak K, Beck F, Chenevix-Trench G, Young J, Jass J, Leggett B, Wainwright B (1998). CDX2, a human homologue of Drosophila caudal, is mutated in both alleles in a replication error positive colorectal cancer. Oncogene.

[B23] Schlichtholz B, Legros Y, Gillet D, Gaillard C, Marty M, Lane D, Calvo F, Soussi T (1992). The immune response to p53 in breast cancer patients is directed against immunodominant epitopes unrelated to the mutational hot spot. Cancer Res.

[B24] Soussi T (2000). p53 Antibodies in the sera of patients with various types of cancer: a review. Cancer Res.

[B25] Brass N, Racz A, Bauer C, Heckel D, Sybrecht G, Meese E (1999). Role of amplified genes in the production of autoantibodies. Blood.

[B26] Cancer Immunome Database. http://www2.licr.org/CancerImmunomeDB/.

[B27] Platzer A, Perco P, Lukas A, Mayer B (2007). Characterization of protein-interaction networks in tumors. BMC Bioinformatics.

[B28] Bignell G, Smith R, Hunter C, Stephens P, Davies H, Greenman C, Teague J, Butler A, Edkins S, Stevens C, O'Meara S, Parker A, Avis T, Barthorpe S, Brackenbury L, Buck G, Clements J, Cole J, Dicks E, Edwards K, Forbes S, Gorton M, Gray K, Halliday K, Harrison R, Hills K, Hinton J, Jones D, Kosmidou V, Laman R, Lugg R, Menzies A, Perry J, Petty R, Raine K, Shepherd R, Small A, Solomon H, Stephens Y, Tofts C, Varian J, Webb A, West S, Widaa S, Yates A, Gillis AJ, Stoop HJ, van Gurp RJ, Oosterhuis JW, Looijenga LH, Futreal PA, Wooster R, Stratton MR (2006). Sequence analysis of the protein kinase gene family in human testicular germ-cell tumors of adolescents and adults. Genes Chromosomes Cancer.

[B29] Davies H, Hunter C, Smith R, Stephens P, Greenman C, Bignell G, Teague J, Butler A, Edkins S, Stevens C, Parker A, O'Meara S, Avis T, Barthorpe S, Brackenbury L, Buck G, Clements J, Cole J, Dicks E, Edwards K, Forbes S, Gorton M, Gray K, Halliday K, Harrison R, Hills K, Hinton J, Jones D, Kosmidou V, Laman R, Lugg R, Menzies A, Perry J, Petty R, Raine K, Shepherd R, Small A, Solomon H, Stephens Y, Tofts C, Varian J, Webb A, West S, Widaa S, Yates A, Brasseur F, Cooper CS, Flanagan AM, Green A, Knowles M, Leung SY, Looijenga LH, Malkowicz B, Pierotti MA, Teh BT, Yuen ST, Lakhani SR, Easton DF, Weber BL, Goldstraw P, Nicholson AG, Wooster R, Stratton MR, Futreal PA (2005). Somatic mutations of the protein kinase gene family in human lung cancer. Cancer Res.

[B30] von Mensdorff-Pouilly S, Gourevitch MM, Kenemans P, Verstraeten AA, van Kamp GJ, Kok A, van Uffelen K, Snijdewint FG, Paul MA, Meijer S, Hilgers J (1998). An enzyme-linked immunosorbent assay for the measurement of circulating antibodies to polymorphic epithelial mucin (MUC1). Tumour Biol.

[B31] Kim JH, Herlyn D, Wong KK, Park DC, Schorge JO, Lu KH, Skates SJ, Cramer DW, Berkowitz RS, Mok SC (2003). Identification of epithelial cell adhesion molecule autoantibody in patients with ovarian cancer. Clin Cancer Res.

[B32] Mosolits S, Harmenberg U, Ruden U, Ohman L, Nilsson B, Wahren B, Fagerberg J, Mellstedt H (1999). Autoantibodies against the tumour-associated antigen GA733-2 in patients with colorectal carcinoma. Cancer Immunol Immunother.

[B33] Ho M, Hassan R, Zhang J, Wang QC, Onda M, Bera T, Pastan I (2005). Humoral immune response to mesothelin in mesothelioma and ovarian cancer patients. Clin Cancer Res.

[B34] Luborsky JL, Barua A, Shatavi SV, Kebede T, Abramowicz J, Rotmensch J (2005). Anti-tumor antibodies in ovarian cancer. Am J Reprod Immunol.

[B35] Le Naour F, Brichory F, Misek DE, Brechot C, Hanash SM, Beretta L (2002). A distinct repertoire of autoantibodies in hepatocellular carcinoma identified by proteomic analysis. Mol Cell Proteomics.

[B36] Gautier F, Irminger-Finger I, Gregoire M, Meflah K, Harb J (2000). Identification of an apoptotic cleavage product of BARD1 as an autoantigen: a potential factor in the antitumoral response mediated by apoptotic bodies. Cancer Res.

[B37] Jiang H, Feng Y (2006). Hypoxia-inducible factor 1alpha (HIF-1alpha) correlated with tumor growth and apoptosis in ovarian cancer. Int J Gynecol Cancer.

[B38] Johnson DG, Cress WD, Jakoi L, Nevins JR (1994). Oncogenic capacity of the E2F1 gene. Proc Natl Acad Sci U S A.

[B39] Rhodes DR, Kalyana-Sundaram S, Mahavisno V, Barrette TR, Ghosh D, Chinnaiyan AM (2005). Mining for regulatory programs in the cancer transcriptome. Nat Genet.

[B40] Seth A, Papas TS (1990). The c-ets-1 proto-oncogene has oncogenic activity and is positively autoregulated. Oncogene.

[B41] Kanehisa M, Goto S, Hattori M, Aoki-Kinoshita KF, Itoh M, Kawashima S, Katayama T, Araki M, Hirakawa M (2006). From genomics to chemical genomics: new developments in KEGG. Nucleic Acids Res.

[B42] Brown KR, Jurisica I (2005). Online predicted human interaction database.. Bioinformatics.

[B43] Schaner ME, Ross DT, Ciaravino G, Sorlie T, Troyanskaya O, Diehn M, Wang YC, Duran GE, Sikic TL, Caldeira S, Skomedal H, Tu IP, Hernandez-Boussard T, Johnson SW, O'Dwyer PJ, Fero MJ, Kristensen GB, Borresen-Dale AL, Hastie T, Tibshirani R, van de Rijn M, Teng NN, Longacre TA, Botstein D, Brown PO, Sikic BI (2003). Gene expression patterns in ovarian carcinomas. Mol Biol Cell.

[B44] Welsh JB, Zarrinkar PP, Sapinoso LM, Kern SG, Behling CA, Monk BJ, Lockhart DJ, Burger RA, Hampton GM (2001). Analysis of gene expression profiles in normal and neoplastic ovarian tissue samples identifies candidate molecular markers of epithelial ovarian cancer. Proc Natl Acad Sci U S A.

[B45] Uhlen M, Bjorling E, Agaton C, Szigyarto CA, Amini B, Andersen E, Andersson AC, Angelidou P, Asplund A, Asplund C, Berglund L, Bergstrom K, Brumer H, Cerjan D, Ekstrom M, Elobeid A, Eriksson C, Fagerberg L, Falk R, Fall J, Forsberg M, Bjorklund MG, Gumbel K, Halimi A, Hallin I, Hamsten C, Hansson M, Hedhammar M, Hercules G, Kampf C, Larsson K, Lindskog M, Lodewyckx W, Lund J, Lundeberg J, Magnusson K, Malm E, Nilsson P, Odling J, Oksvold P, Olsson I, Oster E, Ottosson J, Paavilainen L, Persson A, Rimini R, Rockberg J, Runeson M, Sivertsson A, Skollermo A, Steen J, Stenvall M, Sterky F, Stromberg S, Sundberg M, Tegel H, Tourle S, Wahlund E, Walden A, Wan J, Wernerus H, Westberg J, Wester K, Wrethagen U, Xu LL, Hober S, Ponten F (2005). A human protein atlas for normal and cancer tissues based on antibody proteomics. Mol Cell Proteomics.

[B46] Berlingieri MT, Pallante P, Guida M, Nappi C, Masciullo V, Scambia G, Ferraro A, Leone V, Sboner A, Barbareschi M, Ferro A, Troncone G, Fusco A (2007). UbcH10 expression may be a useful tool in the prognosis of ovarian carcinomas. Oncogene.

[B47] Hassan R, Remaley AT, Sampson ML, Zhang J, Cox DD, Pingpank J, Alexander R, Willingham M, Pastan I, Onda M (2006). Detection and quantitation of serum mesothelin, a tumor marker for patients with mesothelioma and ovarian cancer. Clin Cancer Res.

[B48] Kupryjanczyk J, Thor AD, Beauchamp R, Merritt V, Edgerton SM, Bell DA, Yandell DW (1993). p53 gene mutations and protein accumulation in human ovarian cancer. Proc Natl Acad Sci U S A.

[B49] van Niekerk CC, Boerman OC, Ramaekers FC, Poels LG (1991). Marker profile of different phases in the transition of normal human ovarian epithelium to ovarian carcinomas. Am J Pathol.

[B50] Wu JY, Vlastos AT, Pelte MF, Caligo MA, Bianco A, Krause KH, Laurent GJ, Irminger-Finger I (2006). Aberrant expression of BARD1 in breast and ovarian cancers with poor prognosis. Int J Cancer.

[B51] Wang V, Li C, Lin M, Welch W, Bell D, Wong YF, Berkowitz R, Mok SC, Bandera CA (2005). Ovarian cancer is a heterogeneous disease. Cancer Genet Cytogenet.

[B52] Hamanaka Y, Suehiro Y, Fukui M, Shikichi K, Imai K, Hinoda Y (2003). Circulating anti-MUC1 IgG antibodies as a favorable prognostic factor for pancreatic cancer. Int J Cancer.

[B53] Rhodes DR, Chinnaiyan AM (2005). Integrative analysis of the cancer transcriptome. Nat Genet.

[B54] Mitchell MS, Kan-Mitchell J, Minev B, Edman C, Deans RJ (2000). A novel melanoma gene (MG50) encoding the interleukin 1 receptor antagonist and six epitopes recognized by human cytolytic T lymphocytes. Cancer Res.

[B55] Takahashi Y, Ishii Y, Nishida Y, Ikarashi M, Nagata T, Nakamura T, Yamamori S, Asai S (2006). Detection of aberrations of ubiquitin-conjugating enzyme E2C gene (UBE2C) in advanced colon cancer with liver metastases by DNA microarray and two-color FISH. Cancer Genet Cytogenet.

[B56] PubMed. http://www.ncbi.nlm.nih.gov/sites/entrez?db=PubMed.

[B57] Karanam S, Moreno CS (2004). CONFAC: automated application of comparative genomic promoter analysis to DNA microarray datasets. Nucleic Acids Res.

[B58] Wingender E, Chen X, Hehl R, Karas H, Liebich I, Matys V, Meinhardt T, Pruss M, Reuter I, Schacherer F (2000). TRANSFAC: an integrated system for gene expression regulation. Nucleic Acids Res.

[B59] Perco P, Kainz A, Mayer G, Lukas A, Oberbauer R, Mayer B (2005). Detection of coregulation in differential gene expression profiles. Biosystems.

[B60] Bader GD, Betel D, Hogue CWV (2003). BIND: the Biomolecular Interaction Network Database.. Nucleic Acids Res.

[B61] Zanzoni A, Montecchi-Palazzi L, Quondam M, Ausiello G, Helmer-Citterich M, Cesareni G (2002). MINT: a Molecular INTeraction database. FEBS Lett.

[B62] Mewes HW, Frishman D, Mayer KF, Munsterkotter M, Noubibou O, Pagel P, Rattei T, Oesterheld M, Ruepp A, Stumpflen V (2006). MIPS: analysis and annotation of proteins from whole genomes in 2005. Nucleic Acids Res.

[B63] Peri S, Navarro JD, Kristiansen TZ, Amanchy R, Surendranath V, Muthusamy B, Gandhi TKB, Chandrika KN, Deshpande N, Suresh S, Rashmi BP, Shanker K, Padma N, Niranjan V, Harsha HC, Talreja N, Vrushabendra BM, Ramya MA, Yatish AJ, Joy M, Shivashankar HN, Kavitha MP, Menezes M, Choudhury DR, Ghosh N, Saravana R, Chandran S, Mohan S, Jonnalagadda CK, Prasad CK, Kumar-Sinha C, Deshpande KS, Pandey A (2004). Human protein reference database as a discovery resource for proteomics.. Nucleic Acids Res.

[B64] Suzuki H, Saito R, Kanamori M, Kai C, Schonbach C, Nagashima T, Hosaka J, Hayashizaki Y (2003). The mammalian protein-protein interaction database and its viewing system that is linked to the main FANTOM2 viewer. Genome Res.

[B65] Chen JY, Shen C, Sivachenko AY (2006). Mining alzheimer disease relevant proteins from integrated protein interactome data.. Pac Symp Biocomput.

[B66] Perco P, Rapberger R, Siehs C, Lukas A, Oberbauer R, Mayer G, Mayer B (2006). Transforming omics data into context: bioinformatics on genomics and proteomics raw data. Electrophoresis.

[B67] Nakai K (2000). Protein sorting signals and prediction of subcellular localization. Adv Protein Chem.

[B68] Nakai K, Kanehisa M (1992). A knowledge base for predicting protein localization sites in eukaryotic cells. Genomics.

[B69] Zhang X, Feng J, Cheng Y, Yao Y, Ye X, Fu T, Cheng H (2005). Characterization of differentially expressed genes in ovarian cancer by cDNA microarrays. Int J Gynecol Cancer.

[B70] De Cecco L, Marchionni L, Gariboldi M, Reid JF, Lagonigro MS, Caramuta S, Ferrario C, Bussani E, Mezzanzanica D, Turatti F, Delia D, Daidone MG, Oggionni M, Bertuletti N, Ditto A, Raspagliesi F, Pilotti S, Pierotti MA, Canevari S, Schneider C (2004). Gene expression profiling of advanced ovarian cancer: characterization of a molecular signature involving fibroblast growth factor 2. Oncogene.

[B71] Donninger H, Bonome T, Radonovich M, Pise-Masison CA, Brady J, Shih JH, Barrett JC, Birrer MJ (2004). Whole genome expression profiling of advance stage papillary serous ovarian cancer reveals activated pathways. Oncogene.

[B72] Lancaster JM, Dressman HK, Whitaker RS, Havrilesky L, Gray J, Marks JR, Nevins JR, Berchuck A (2004). Gene expression patterns that characterize advanced stage serous ovarian cancers. J Soc Gynecol Investig.

[B73] Lee BC, Cha K, Avraham S, Avraham HK (2004). Microarray analysis of differentially expressed genes associated with human ovarian cancer. Int J Oncol.

[B74] Santin AD, Zhan F, Bellone S, Palmieri M, Cane S, Bignotti E, Anfossi S, Gokden M, Dunn D, Roman JJ, O'Brien TJ, Tian E, Cannon MJ, Shaughnessy J, Pecorelli S (2004). Gene expression profiles in primary ovarian serous papillary tumors and normal ovarian epithelium: identification of candidate molecular markers for ovarian cancer diagnosis and therapy. Int J Cancer.

[B75] Collins Y, Tan DF, Pejovic T, Mor G, Qian F, Rutherford T, Varma R, McQuaid D, Driscoll D, Jiang M, Deeb G, Lele S, Nowak N, Odunsi K (2004). Identification of differentially expressed genes in clinically distinct groups of serous ovarian carcinomas using cDNA microarray. Int J Mol Med.

[B76] Hibbs K, Skubitz KM, Pambuccian SE, Casey RC, Burleson KM, Oegema TR, Thiele JJ, Grindle SM, Bliss RL, Skubitz AP (2004). Differential gene expression in ovarian carcinoma: identification of potential biomarkers. Am J Pathol.

[B77] Adib TR, Henderson S, Perrett C, Hewitt D, Bourmpoulia D, Ledermann J, Boshoff C (2004). Predicting biomarkers for ovarian cancer using gene-expression microarrays. Br J Cancer.

[B78] Zorn KK, Jazaeri AA, Awtrey CS, Gardner GJ, Mok SC, Boyd J, Birrer MJ (2003). Choice of normal ovarian control influences determination of differentially expressed genes in ovarian cancer expression profiling studies. Clin Cancer Res.

[B79] Matei D, Graeber TG, Baldwin RL, Karlan BY, Rao J, Chang DD (2002). Gene expression in epithelial ovarian carcinoma. Oncogene.

[B80] Sawiris GP, Sherman-Baust CA, Becker KG, Cheadle C, Teichberg D, Morin PJ (2002). Development of a highly specialized cDNA array for the study and diagnosis of epithelial ovarian cancer. Cancer Res.

[B81] Shridhar V, Lee J, Pandita A, Iturria S, Avula R, Staub J, Morrissey M, Calhoun E, Sen A, Kalli K, Keeney G, Roche P, Cliby W, Lu K, Schmandt R, Mills GB, Bast RC, James CD, Couch FJ, Hartmann LC, Lillie J, Smith DI (2001). Genetic analysis of early- versus late-stage ovarian tumors. Cancer Res.

[B82] Hough CD, Cho KR, Zonderman AB, Schwartz DR, Morin PJ (2001). Coordinately up-regulated genes in ovarian cancer. Cancer Res.

[B83] Mok SC, Chao J, Skates S, Wong K, Yiu GK, Muto MG, Berkowitz RS, Cramer DW (2001). Prostasin, a potential serum marker for ovarian cancer: identification through microarray technology. J Natl Cancer Inst.

[B84] Hough CD, Sherman-Baust CA, Pizer ES, Montz FJ, Im DD, Rosenshein NB, Cho KR, Riggins GJ, Morin PJ (2000). Large-scale serial analysis of gene expression reveals genes differentially expressed in ovarian cancer. Cancer Res.

[B85] Ismail RS, Baldwin RL, Fang J, Browning D, Karlan BY, Gasson JC, Chang DD (2000). Differential gene expression between normal and tumor-derived ovarian epithelial cells. Cancer Res.

[B86] Ono K, Tanaka T, Tsunoda T, Kitahara O, Kihara C, Okamoto A, Ochiai K, Takagi T, Nakamura Y (2000). Identification by cDNA microarray of genes involved in ovarian carcinogenesis. Cancer Res.

[B87] Schummer M, Ng WV, Bumgarner RE, Nelson PS, Schummer B, Bednarski DW, Hassell L, Baldwin RL, Karlan BY, Hood L (1999). Comparative hybridization of an array of 21,500 ovarian cDNAs for the discovery of genes overexpressed in ovarian carcinomas. Gene.

